# A Bankruptcy Problem Approach to Load-shedding in Multiagent-based Microgrid Operation

**DOI:** 10.3390/s101008888

**Published:** 2010-09-28

**Authors:** Hak-Man Kim, Tetsuo Kinoshita, Yujin Lim, Tai-Hoon Kim

**Affiliations:** 1 Department of Electrical Engineering, University of Incheon / 12-1, Sondo-dong, Yeonsu-gu, Incheon, 406-840, Korea; E-Mail: hmkim@incheon.ac.kr; 2 Graduate School of Information Science, Tohoku University / 2-1-1, Katahira, Aoba-ku, Sendai, 980-8577, Japan; E-Mail: kino@riec.tohoku.ac.jp; 3 Department of Information Media, University of Suwon / San 2-2, Wau-ri, Bongdam-eup, Hwaseong-si, Gyeonggi-do, 445-743, Korea; 4 Department of Multimedia Engineering, Hannam University / 133, Ojeong-dong, Daedeok-gu, Daejeon, 306-791, Korea; E-Mail: taihoonn@empas.com

**Keywords:** bankruptcy problem, load-shedding, microgrid, islanded operation, multiagent system

## Abstract

A microgrid is composed of distributed power generation systems (DGs), distributed energy storage devices (DSs), and loads. To maintain a specific frequency in the islanded mode as an important requirement, the control of DGs’ output and charge action of DSs are used in supply surplus conditions and load-shedding and discharge action of DSs are used in supply shortage conditions. Recently, multiagent systems for autonomous microgrid operation have been studied. Especially, load-shedding, which is intentional reduction of electricity use, is a critical problem in islanded microgrid operation based on the multiagent system. Therefore, effective schemes for load-shedding are required. Meanwhile, the bankruptcy problem deals with dividing short resources among multiple agents. In order to solve the bankruptcy problem, division rules, such as the constrained equal awards rule (CEA), the constrained equal losses rule (CEL), and the random arrival rule (RA), have been used. In this paper, we approach load-shedding as a bankruptcy problem. We compare load-shedding results by above-mentioned rules in islanded microgrid operation based on wireless sensor network (WSN) as the communication link for an agent’s interactions.

## 1. Introduction

The microgrid, which is an eco-friendly small-scale power grid, is expected to be introduced into power grids in the near future. Especially, the main energy sources of the microgrid are composed of solar and wind power facilities, micro-turbines and fuel cell-based combined heat and power (CHP) systems, and battery devices. The use of these energy sources have been encouraged in many counties because as clean energy sources they have a close relationship with Climate Change. Research and demonstration projects for microgrids have been pursed [1,2].

Since the microgrid is composed of small-scale power systems and devices, autonomic and autonomous microgrids based on the multiagent system have been studied for their economical and efficient operation and control [3–6]. The multiagent system is a society or system composed of multiple artificial agents. The agent has some abilities such as reactivity, pro-activeness, and social ability [7]. The agent is able to control and to operate adaptively and autonomously his/her local system according to the intension of design purpose after sensing environment changes like a human operator.

One of most important requirements of a microgrid operation is to maintain a specific frequency such as 50 Hz or 60 Hz. In the islanded operation mode, which is an operation mode when the microgrid is electrically isolated from power grids, regulation of DGs, charge and discharge actions of DSs, and load-shedding are generally used. Especially, load-shedding, which intentionally reduces electricity use when power supply is short, is a critical problem because the load-shedding is inconvenient to consumers. Therefore, efficient schemes for handling load-shedding are required.

Meanwhile, the bankruptcy problem deals with how to divide an insufficient estate among all claimants and is used in finance, economics, and engineering areas as a subarea of game theory as well as mathematics. To solve bankruptcy problems, various methods such as division rules and cooperative game theories have been studied [8–10].

In this paper, we approach load-shedding in a multiagent-based islanded microgrid as a bankruptcy problem because the purpose of load-shedding is to reduce loads intentionally and divide the reduced amount of electricity among consumers (loads). We consider popular division rules such as the constrained equal awards rule (CEA), the constrained equal losses rule (CEL), and the random arrival rule (RA). Especially, we deal with operation planning of the islanded microgrid, unlike previous works dealing with control of the islanded microgrid [11].

The remainder of this paper is structured as follows. Section 2 describes multiagent-based islanded microgrid operation. Section 3 explains load-shedding schemes based on the bankruptcy problem and division rules. Section 4 shows design and construction of an experimental multiagent-based islanded microgrid. Following this, we test the feasibility of suggested load-shedding schemes based on the divide rules and discuss the feature of the applications in Section 5. Finally, Section 6 summarizes our study results and future plans.

## 2. Multiagent-based Islanded Microgrid Operation

### 2.1. Microgrid Operation

The microgrid is composed of distributed generation systems (DSs), such as solar and wind power facilities, micro-turbines and fuel cell-based CHP systems, and distributed energy storage devices (DSs), such as battery systems and flywheel energy storage devices, and loads.

The microgrid can be operated in the the grid-connected mode and the islanded mode. A Microgrid Operation & Control Center (MGOCC) manages microgrid operation [5,6]. One of important operational requirements is to maintain a specific frequency such as 50 Hz or 60 Hz. The maintenance of the frequency is closely related to a power balance between supply and demand. The frequency increases in supply excess conditions but decreases in supply shortage conditions. To maintain the frequency in the grid-connected mode, the microgrid trades power with the connected power grid. On the other hand, the microgrid intentionally controls supply and demand without any trade with the power grid in the islanded mode because the microgrid is isolated from any power grids. Especially, intentional reduction of demand (or load), which is called to load-shedding, is inconvenient for consumers and therefore, is a critical problem.

**Figure 1.**
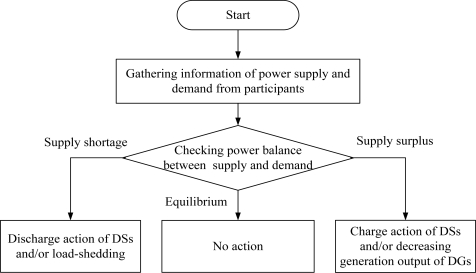
Typical operation for the islanded mode.

In the case of one-owner operation or fully authorized operation from all participants in the microgrid, load-shedding is performed according to a priority order based on load importance. However, in the case of an energy market environment having multiple independent participants, load-shedding should be performed by some reasonable scheme excluding critical loads agreed by all participants. Figure 1 shows a typical operation for the islanded mode.

**Figure 2.**
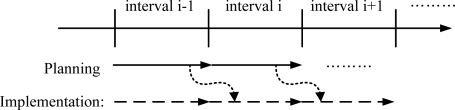
Microgrid operation procedure.

In this paper, we assume that microgrids are operated in two steps: planning and implementation, as shown in Figure 2 [5,6]. The MGOCC should establish an operation plan for the next interval and should implement the operation plan established during the previous interval. The interval period depends on microgrid operation rules in general.

### 2.2. Multiagent System

**Figure 3.**
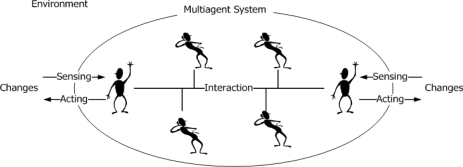
Multiagent system.

An agent is able to sense changes of the environment and to act autonomously according to the intension of design purpose. Multiagent system is a system or society composed of multiple agents. In the multiagent system, agents communicate with an agent communication language (ACL) and share knowledge for cooperation. Figure 3 shows the multiagent system.

### 2.3. Multiagent-Based Microgrid

A multiagent-based microgrid is a microgrid operated by autonomous agents. A multiagent system for microgrid operation is defined as follows [5,6]:

(1) $$\mathrm{Ag}=\left\{{\mathrm{Ag}}_{\mathrm{MGOCC}},{\mathrm{AG}}_{\mathrm{DG}},{\mathrm{AG}}_{\mathrm{DS}},{\mathrm{AG}}_{L}\right\}$$

where *Ag_MGOCC_* is the MGOCC agent, *AG_DG_* is a set of DG agents (*Ag_DG_*), *AG_DS_* is a set of storage device agents (*Ag_DS_*), and *AG_L_* is a set of load agents (*Ag_L_*). The MOGCC agent manages the entire operation of the microgrid. Each agent controls and operates his/her DG, DS or load.

**Figure 4.**
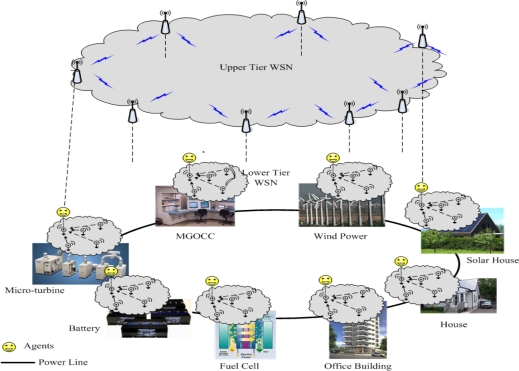
Multiagent-based microgrid based on WSN.

Figure 4 shows a multiagent-based microgrid. In this microgrid, in order to reduce the deployment and management costs, a wireless sensor network (WSN) is considered as the communication link for agents’ interactions. In order to achieve reliable levels of distributed control of the microgrid, it is imperative that the microgrid possesses a self configurable WSN that aids in communication among agents. We consider two-tier network architecture for the microgrid, as presented in Figure 4. The lower layer is the WSN that is used for autonomous communication among the metering devices and various appliance control modules. The primary design goals for this layer include short range in-building operation and self-healing via topological reconfiguration for reliable operation. Candidate wireless technologies are IEEE 802.11n, IEEE 802.15.4, and Bluetooth. The upper layer corresponds to the mesh WSN used for intra-microgrid control by enabling information exchanges between agents. The main requirements of design include supporting the multi-point to multi-point data model, and fault tolerance for reliability. Networking technologies such as IEEE 802.11n and IEEE 802.16 can be used for forming links between agents.

## 3. Load-shedding Based on the Bankruptcy Problem

### 3.1. Mathematic Expression of Load Shedding Based on Bankruptcy Problem

The bankruptcy problem deals with how to divide an insufficient estate among all claimants. In this paper, we approach the load-shedding problem as a bankruptcy problem. The load-shedding problem can be defined as a pair (*lc, Pa*), where *Pa* is available power and *lc* = (*lc_1_, ······, lc_n_*) is the vector of load claims. *Pa* and *lc* are described as:

(2) $$0\leq {\mathrm{lc}}_{1}\leq \cdot \cdot \cdot \cdot \cdot \cdot \leq {\mathrm{lc}}_{n}\;\text{and}\;0\leq \mathrm{Pa}\leq {\mathrm{lc}}_{1}+\cdot \cdot \cdot \cdot \cdot \cdot +{\mathrm{lc}}_{n}$$

The vector of allocated power (la*) of each load is defined as:

(3) $$\mathrm{la}*=\left({\mathrm{la}}_{1}*,\cdot \cdot \cdot \cdot \cdot \cdot ,{\mathrm{la}}_{n}*\right)$$

Finally, the vector of load-shedding of each load (ls*) is defined as:

(4) $$\mathrm{ls}*=\left({\mathrm{ls}}_{1}*,\cdot \cdot \cdot \cdot \cdot \cdot ,{\mathrm{ls}}_{n}*\right)=\mathrm{lc}-\mathrm{la}*$$

### 3.2. Load-Shedding Schemes Based on Division Rules

In this paper, we consider the well-known CEA, the CEL and the RA as algorithms for the bankruptcy problem. The details of the rules are described in [9]. To allocate short power to loads, we define the following load-shedding rule based on the CEA:

(5) $${\mathrm{CEA}}_{i}(\mathrm{lc},\mathrm{Pa})=\mathrm{la}*=\text{min}\{{\mathrm{lc}}_{i},\lambda \}$$

where, *λ* is chosen so that Σ min{*lc_j_*, *λ*} = *Pa*.

In order to allocate short power to loads using the CEL, we define the following rule:

(6) $${\mathrm{CEL}}_{i}\left(\mathrm{lc},\mathrm{Pa}\right)=\mathrm{la}*=\text{max}\left\{0,{\mathrm{lc}}_{i}-\lambda \right\}$$

where, *λ* is chosen so that Σ max{*0*, *lc_j_* − *λ*} = *Pa*.

To allocate short power to loads, we define the following rule based on the RA:

(7) $${\mathrm{RA}}_{i}\left(\mathrm{lc},\mathrm{Pa}\right)=\mathrm{la}*=\frac{1}{n!}\text{min}\left\{{\mathrm{lc}}_{i},\text{max}\left\{\mathrm{Pa}-\sum_{j\in N,\pi (j)<\pi (i)}{\mathrm{lc}}_{j},0\right\}\right\}$$

**Figure 5.**
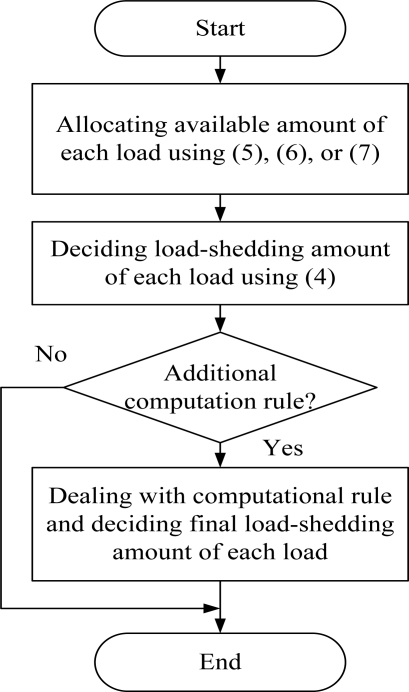
Load-shedding procedure.

Finally, load-shedding is decided by the above-mentioned equation (4). Figure 5 shows load-shedding schemes based on division rules, where we consider additional computation rule for practical applications; *Computation Rule*) When there are more two digits below the decimal point, the exceed digits are truncated and added to a consumer requiring the largest load amount.

## 4. Building Experimental Multiagent-Based Microgrid

### 4.1. Design of Interactions

A multiagent system, which is defined by above-mentioned equation (1), is used for tests. The MGOCC agent is a manager agent for microgrid operation. For interactions among the agents, we use two communication protocols as follows:

- Information Exchange Protocol for interactions between *Ag_MGOCC_* and *AG_DS_*, *AG_L_*
- Modified Contract Net Protocol (CNP) for interactions between *Ag_MGOCC_* and *AG_DG_*.

We employ a modified version of the Knowledge Query and Manipulation Language (KQML) as the agent communication language (ACL) and use the following message format for cooperation among agents [12]:

$$\begin{array}{c} (<\text{performative}>\;:\text{from}\;<\text{agent}\;\text{name}>\;:\text{to}\;<\text{agent}\;\text{name}>\\ :\text{content}\;<\text{OAV}\;\text{type}\;\text{data}>). \end{array}$$

**Figure 6.**
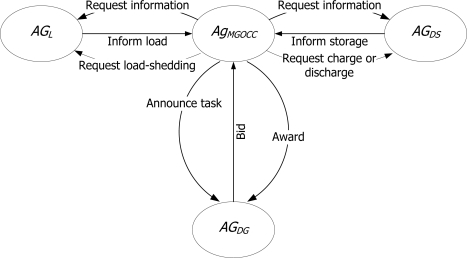
Message flow.

Figure 6 shows designed message flow among the agents for interactions. In this figure, we can see the modified CNP as a protocol between the MGOCC agents and DG agents and the Information Exchange Protocol for interactions between the MGOCC agents and DS agents/load agents.

We use a state function (*T*) for effective task management as follows:

(7) $$\left(s^{\prime },a\right)=T\left(s,e\right)$$

where s’, a, s and e mean the new state, the action, the current state, and the event, respectively [12].

In the operation procedure based on the multiagent system, the final contractors among DGs are decided by the merit order algorithm [5].

### 4.2. Implementation

We implement our multiagent system for microgrid operation with the Distributed Agent System based on Hybrid Architecture (DASH), the Interactive Design Environment for Agent Designing Framework (IDEA), and the Java, where the DASH is a multiagent platform, the IDEA is a GUI-based interactive environment for the DASH platform, and the Java is used for user-defined functions [5,13–15].

## 5. Experiment

### 5.1. Test and Results

In this experiment, we compare the feature of load-shedding based on the CEA, the CEL, and the RA. For this, the following multiagent-based islanded microgrid is constructed.

- Agents: the MGOCC agent, three DG agents, a DS agent, ten Load agents.
- Capacity of DS: 10 [kWh]
- Initial charged amount of DS: 5 [kWh]
- L1: 30 [kWh]
- L2: 50 [kWh]
- L3: 60 [kWh]
- L4: 100 [kWh]
- L5: 110 [kWh]
- L6: 120 [kWh]
- L7: 150 [kWh]
- L8: 200 [kWh]
- L9: 230 [kWh]
- L10: 250 [kWh]
- Total load amount: 1,300 [kWh]
- Supply shortage by three DGs: from 1,100 [kWh] to 100 [kWh] with decrease by 200 [kWh] in 6 cases

**Figure 7.**
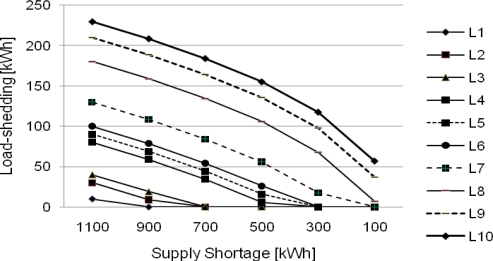
Load-shedding based on the CEA.

**Figure 8.**
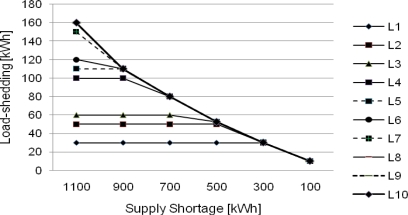
Load-shedding based on the CEL.

**Figure 9.**
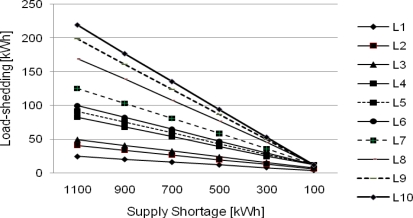
Load-shedding based on the RA.

Figures 7, 8, and 9 show results of load-shedding using the CEA, the CEL, and the RA, respectively. In the CEA application as shown in Figure 7, equal power is allocated to all load agents in a serious power shortage of 1,100 kWh. However, in the case of small supply shortage, Load agents claiming a lot of power take charge of load-shedding and load agents claiming small power are ruled out from load-shedding.

In the CEL application, as shown in Figure 8, equal load-shedding is applied to load agents not limited by their power claims and amounts of load claims are imposed to load agents limited by their power claims as load-shedding amounts in power shortage of from 1,100 kWh to 300 kWh in the figure. In power shortage of from 300 kWh to 100 kWh, equal load-shedding is applied to each load agent, where load-shedding amount imposed evenly to each load agent is less than or equal to the load amount of L1, which is smallest load.

In the RA application, as shown in Figure 9, load-shedding is performed to be proportional to load amount claimed by each Load agent within power claim of each load agent.

### 5.2. Discussion

In this experiment, we can see different patterns of load-shedding by the CEA, the CEL, and the RA. Table 1 shows the feature of three load-shedding schemes. In general, supply shortages are not serious in the islanded microgrid because power generation systems are installed considering the total amount of loads in the islanded operation mode. Therefore, small supply shortages are anticipated under supply shortage conditions. If we take into account the small supply shortages, the feature of load-shedding based on the division rules is summarized as follows:

- The CEA has a merit on operational convenience because load-shedding is only required to some loads having a lot of power claim when we consider that exact regulation of load by a control reference plan is not easy.
- The CEL and the RA have a merit on fairness of load-shedding because of load-shedding is required to all loads evenly and proportionally, respectively.

To select best rule for load-shedding is not easy because the CEA has the merit of operational convenience and the CEL and the RA have the merits of fairness of load-shedding as mentioned above. The choice of a load-shedding scheme for an islanded microgrid should depend on an agreement by all participants considering the above-mentioned features according to the three load-shedding schemes in principle. Realistically, if we consider that the precise regulation of load amount is not easy, the CEA can be recommended, even though the CEA is less fair than either the CEL and the RA schemes.

## 6. Conclusions

This paper presents load-shedding schemes based on the bankruptcy problem and division rules in islanded microgrid operation. The contributions of this paper are stated as follows:

1. Load-shedding is approached as a bankruptcy problem.
2. In order to decide load-shedding, the CEA, the CEL, and the RA are considered as candidate division rules.
3. Load-shedding schemes based on the above-mentioned three division rules are proposed.
4. The load-shedding schemes are tested in the multiagent-based microgrid based on wireless sensor network (WSN) as a communication link for agents’ interactions of the islanded microgrid.
5. The feature of load-shedding schemes is illustrated and discussed. From the experiment, the CEA has merit in operational convenience and the CEL and the RA have the merit of fairness of load-shedding.

In our work, we considered three well-known division rules for load-shedding applications. As a further work, we are considering game theory approaches for load-shedding in islanded microgrid operation.

## Figures and Tables

**Table 1.** The feature of three load-shedding schemes.

**Load-shedding scheme**	**Serious supply shortage**	**Small supply shortage**
CEA	• Equal load allocation	• Small loads are ruled out
CEL	• Equal load-shedding within claiming load	• Equal Load-shedding
RA	• Proportional load-shedding within claiming load	• Proportional load-shedding within claiming load
